# Goal management training improves executive control in adults with ADHD: an open trial employing attention network theory to examine effects on attention

**DOI:** 10.1186/s40359-022-00902-9

**Published:** 2022-08-26

**Authors:** Daniel A. Jensen, Astri J. Lundervold, Jan Stubberud, Anne Halmøy, Jan Haavik, Lin Sørensen

**Affiliations:** 1 Department of Biological and Medical Psychology, University of Bergen, Bergen, Norway; 2 Division of Mental Health, Betanien Hospital, Bergen, Norway; 3 Department of Psychology, University of Oslo, Oslo, Norway; 4 Department of Research, Lovisenberg Diaconal Hospital, Oslo, Norway; 5 Division of Psychiatry, Haukeland University Hospital, Bergen, Norway; 6 Department of Clinical Medicine, University of Bergen, Bergen, Norway; 7 Department of Biomedicine, University of Bergen, Bergen, Norway

**Keywords:** Attention deficit disorder with hyperactivity, Cognitive control, Executive function, Treatment, Cognitive rehabilitation

## Abstract

**Background:**

Adults with Attention-Deficit/Hyperactivity Disorder (ADHD) typically experience poorer attentional control. According to the attention network theory, attentional control relies on three interacting networks of alerting, orienting, and executive control. In ADHD, it is mainly the alerting and executive control networks that are suggested and found to be compromised.

**Methods:**

In the current study, we investigated if a group-based metacognitive remediation program (Goal Management Training [GMT]) in adults with ADHD would enhance attentional control using an experimental measure of the attention network theory. We expected that GMT would specifically enhance the executive control and alerting networks.

**Results:**

Data from post- and follow up-assessments of 21 adults (age: 39.05 [11.93]) with ADHD who had completed GMT were included. Linear mixed-effects modeling revealed significant improvements in the functioning of the executive control network for the majority of the participants, although a small subset of participants showed a negative development following the intervention. Results also showed an improvement in the orienting network at follow up, but no change in the alerting network.

**Conclusion:**

The results may indicate that improvements in the functioning of the executive control network are central to the positive effects of GMT reported in disorders characterized by impaired attentional control.

*Trial registration*: The study was retrospectively registered in the ISRCTN (Identifier: ISRCTN91988877) on the 18/01/2021.

**Supplementary Information:**

The online version contains supplementary material available at 10.1186/s40359-022-00902-9.

## Introduction

Adults with Attention-Deficit/Hyperactivity Disorder (ADHD) struggle with elevated symptoms of inattention and/or hyperactivity/impulsivity in their everyday life [1]. Typically, this is reflected in a poorer ability to voluntarily focus their attention on the task at hand and maintain this attentional focus over time to aid goal-directed behavior [2–6]. As a result, they often experience academic [7–11] and occupational [9, 12–14] impairments. In ADHD, pharmacological treatment has been shown to reduce alterations in brain activity and structures in brain networks involved in attentional control [6]. However, pharmacological therapy has limited efficacy and not all individuals with ADHD tolerate such treatment [15]. Efforts have been made to investigate if it is possible to train attentional control and thereby enhance attention functions. Still, there is limited evidence for the effectiveness of non-pharmacological interventions for ADHD [15] including interventions focusing on attentional control [16]. The aim of the current study was therefore to investigate the effectiveness of a group-based metacognitive remediation program, Goal Management Training (GMT; [17, 18], in enhancing attentional control in adults with ADHD.

GMT is a group-based metacognitive remediation program aimed at reducing deficiencies of goal management, based on Duncan’s [19] attentional control theory of goal neglect [17, 18]. The theory describes how goal management fails when an individual is unable to maintain task focus towards future goals due to being unable to cope with the competing demands of other salient stimuli or ongoing activities. The program, therefore, emphasizes teaching participants a five-stage strategy to increase goal attainment. The intervention builds on the assumption that participants, through practice, will be able to improve sustained attention via changes in the brain networks underlying this function and that this will allow for improved executive control [20]. Important components include the intermittent stopping of ongoing behavior (“STOP!-and-think”) to orient towards relevant goals and to evaluate whether ongoing behavior is in line with these. The program also contains components of mindfulness training [21] meant to help participants develop the skills needed to maintain a focus on the present, as well as the active use of self-cueing to regulate alertness to maintain executive control [22]). These strategies and techniques are combined with the aim of improving awareness of attentional lapses to reduce the negative influence of poorer executive control. Thus, GMT can be said to target executive control and attention allocation [20].

GMT has been shown to have positive effects on measures of executive control and attention in several patient groups [23, 24]. However, to the best of our knowledge, only two earlier, small-scale pilot studies have investigated the effects of GMT in adults with ADHD [25, 26]. In de Braek et al. reported positive effects of GMT compared to psychoeducation with regards to clinician-rated, everyday cognitive functioning. However, they only included a general performance-based measure of problem solving, and no performance-based measures that specifically tap into executive control and attention allocation. Jensen et al., studying the same adult ADHD sample as in the current study, reported significant improvements on measures of executive control, such as on the Stroop test and the Tower test following GMT. However, these measures do not allow for differentiation between executive control and attention allocation of alertness and orienting attention [26]. Applying a systems-neuroscience approach for measuring effects of GMT would improve the understanding of which specific attention function(s) GMT improves in adults with ADHD.

The attention network theory [27–29] offers such a systems-neuroscience approach, defining three interacting networks of early-operating attention allocation of alerting and orienting, as well as executive control [27, 29–31]. The executive control network is involved in complex operations, such as detecting and resolving conflicts between stimuli, for instance in inhibiting salient stimuli to be able to attend to a task-based target stimulus. To facilitate executive control, the alerting network supports preparation for shifting from rest to task-based activity and to maintain effort over time (alertness). The orienting network, on the other hand, is involved in the selection of which stimuli to attend among multiple stimuli, such as the ability to quickly focus attention on task-based stimuli. ANT has not previously been applied in studies of GMT [23]. However, Berger and Posner [30] hypothesized that individuals with ADHD would show poorer functioning of the attention networks of executive control and alerting but not the orienting network. This has been supported in studies of children [32–36] and adults with ADHD [37], although there are also contradictory findings from studies of children [39–41]. So far, the use of the attention network theory to examine treatment effects in studies of adults with ADHD is limited. One study by Dotare et al. [42] reported positive effects of a computer-based cognitive remediation training program on executive control and not on the alerting or orienting networks. This is in line with findings showing that children without ADHD experienced positive effects of a tailored computer-based training program [43] specifically on the executive control network, and that children with the poorest attentional control at baseline were most likely to benefit from such interventions. These findings are also comparable to results from studies in which the attention network theory has been applied to test treatment effects in non-ADHD adult samples, such as effects of mindfulness training on attention. In mindfulness training, positive effects have been observed on executive control in addition to the orienting network, whereas positive effects on alerting have only been observed in experienced meditators/mindfulness practitioners [44, 45].

In the present study, we investigated the effects of GMT [17, 18, 46] on the three attention networks as described in the attention network theory [27], using the revised Attention Network Test (ANT-R; 47) in an open trial in which adults with ADHD participated. Since no prior study has tested the effects of GMT with ANT-R, we build on the theory of GMT and previous findings in ADHD samples using the original Attention Network Test [48] to develop the following hypotheses:

We expected (1) that adults with ADHD would show improvements in the executive control and alerting networks following GMT and not on the orienting network. (2) To find the most consistent change in the executive control network since goal achievement through efficient conflict detection is emphasized as a main mechanism in GMT [20]. In addition to the traditional focus on averaged group scores to measure effects of GMT, we also analyzed change scores for every participant to better understand individual differences in effects of GMT.

## Methods

### Participants

Participants in the current study were part of an exploratory investigation of GMT as an intervention for adults with ADHD recruited from local outpatient clinics in the municipality of Bergen, Norway (see 26 for further details). Inclusion criteria were an age > 18 years and a confirmed clinical diagnosis of ADHD. The participants had been diagnosed by a specialist (i.e., clinical psychologist or psychiatrist) outside of the project according to the current Norwegian guidelines [49] which employ diagnostic codes from the ICD-10 [50] but allow for the use of DSM-IV/5 criteria [1, 51] in the diagnostic assessment. These guidelines describe the necessity of assessing developmental history, current symptoms and effects on functioning across multiple domains, and an assessment of physical or psychiatric illnesses that may explain the symptoms. The guidelines recommend the use of the Diagnostic Interview for Adult ADHD, second edition (DIVA 2.0), the Mini International Neuropsychological Interview (M.I.N.I. Plus) and the Structured Clinical Interview II (SCID-II) for DSM-IV axis I and axis II disorders, respectively [52–54], in addition to self-report forms such as the Adult ADHD Self-report Scale (ASRS; [55]) and the Wender-Utah Rating scale (WURS; [56]). These guidelines also emphasize the need to collect collateral reports (e.g., the use of DIVA with parents/older siblings and/or long-term partner/spouse). Exact data on adherence to these guidelines was not available for inclusion in this study. Exclusion criteria in our study sample were a full-scale intelligence quotient below 80, a history of psychotic disorder, or other severe ongoing psychiatric disorders such as severe depression or acute suicidality, which would prohibit participation in the intervention study. In total, 34 participants volunteered for the study of whom 21 participants completed two or more assessment points and were included in the present study. Participants’ age ranged from 21 to 62 years (*M* = 39.05, *SD* = 11.93, 57% males). A total of 13 of these 21 participants (61.9%) fulfilled the criteria for at least one other ongoing disorder according to the M.I.N.I. Plus [54]. Various anxiety disorders constituted the majority of these disorders (Table 1). For further details on recruitment and clinical characterstics of the sample see Table 1 and Jensen et al. [26].

**Table 1:** Descriptive statistics of the sample and included variables

	*n* (%)	*M*	*SD*
Number of males	12 (57.1)		
Number of females	9 (42.9)		
Receiving stimulant medication	10 (47.6)		
Age	21	39.2	11.4
IQ	21	120.1	10.1
Comorbidities			
No other disorder	8 (38.1)		
Major depressive disorder	2 (9.5)		
Anxiety disorder	11 (52.4)		
Other disorders	7 (33.3)		
*ASRS*			
Pre	21	44.4	8.8
Post	20	41.2	9.1
Follow up	20	39.3	11.2
ANT-r measures	21		
*Flanker conflict*			
Pre		175.4	160.7
Post		155.1	147.0
Follow up		150.0	143.6
*Alerting*			
Pre		41.9	175.5
Post		34.7	157.9
*Follow up*		35.0	144.6
Validity			
Pre		93.0	176.0
Post		104.4	162.6
Follow up		112.2	155.1

Receiving medication = number and percentage of participants receiving medication for ADHD at baseline, IQ = full-scale IQ estimate from the Wechsler Abbreviated Scale of Intelligence, Comorbidities = ongoing diagnoses according to M.I.N.I. PLUS, Anxiety disorder = Panic disorder, Agoraphobia, Social phobia or Generalized anxiety disorder, Other disorder = Antisocial personality disorder, Body dysmorphic disorder and PMS dysphoric disorder, ASRS = Sum score from the Adult ADHD Self-report Scale, ANT-*r* measures = calculated effects in milliseconds

### Procedure

Diagnostic assessment of comorbid disorders, self-reports of, among others, ADHD symptoms, and assessments with a neuropsychological test battery and electrocardiogram (ECG) were conducted at the neuropsychological outpatient clinic at the University of Bergen. As participants had an existing ADHD-diagnosis, only self-reports of current symptoms where collected. For further details see Jensen et al. [26]. Participants completed the ANT-R in a soundproof room approximately midway through the assessment and immediately following a break at all three time points.

GMT was administered in nine two-hour group sessions in groups of four to eight participants led by a clinical psychologist (six years of studies at the university level) and a co-therapist who was either a clinical psychologist or a clinical psychology student with clinical experience (i.e., students who had completed at least four and a half of the six years of study and had experience from clinical work as psychologists under supervision). Guidance from a clinical psychologist with extensive experience with GMT was also available. Due to various holidays the duration of the intervention varied between nine and 11 weeks. Participants were asked to complete daily homework assignments between sessions and their experiences with these were discussed in the following session. Participants had to complete a minimum of six out of nine sessions to be included in the post- and follow up-assessments.

Assessments were conducted within three weeks before the first session of the GMT-intervention and within two weeks after the last session. Follow up-assessments were conducted six months after completion of the intervention (± two weeks).

## Measurements

### Attention network test—revised

The ANT-R, developed by Fan et al. [47], is a revision of the original Attention Network Test [48], introducing new elements that allow for the investigation of interactions between the three constituent attentional networks. In the ANT-R the presentation of the flanker condition is presented at one of two locations on a computer screen and these locations can be either congruent or incongruent. Furthermore, the ANT-R includes three cue conditions: no cue, double cue (alerting cues) and spatial cue (orienting cues). In the current study we included the reaction time scores for alerting, orienting and executive control (see Table 2).

**Table 2:** Descriptive information about the ANT-r scores

Attention networks	Variable score	Measures	Operational score calculation
Alerting	Alerting	Tonic^a^ and phasic arousal (temporal cues)	(RT no cue-condition)—(RT double cue-condition)
Orienting	Validity	Endogenous^a^ and exogenous attention engagement (spatial cues)	(RT invalid cue-condition)—(RT valid cue-condition)
Executive control	Flanker conflict	Conflict processing^b^ (congruent and incongruent conditions)	(RT flanker incongruent)—(RT flanker congruent)

*RT*.Reaction time. ^a^Lower scores indicate an effect of the endogenous, self-regulated system on alertness and orienting. ^b^ Lower scores indicate more efficient conflict processing. Table adapted from Sørensen et al. [57]

During performance of the ANT-R, the participants are seated in front of a computer. They are instructed to attend to a fixation crosshair at the center of the screen and informed that a set of five arrows will appear inside one of two boxes which are placed to the left and right of this crosshair. Their task is to indicate the direction of the center arrow as quickly and accurately as possible by pressing the left mouse key with their right index finger if the arrow is facing left, and the right mouse key with their right middle finger if the arrow is facing right. The flanker condition is defined as congruent if the center arrow is facing in the same direction as the surrounding flanker arrows and incongruent if the direction of the flanker arrows is opposite of the direction of the target arrow (e.g., center arrow facing right, flanker arrows facing left). Each trial consists of the presentation of the flanker arrows for 500 ms. This is preceded by the presentation of the varying cue conditions for 100 ms, followed by a cue-target interval of either 0, 400 or 800 ms. During the 100 ms in which the cue is presented the participants receive either no cue, a valid (i.e., the box surrounding the location where the flanker will appear flashes) or invalid (i.e., the box surrounding the location where the flanker will *not* appear flashes) spatial cue, or a temporal cue (both boxes flash). The trials are separated by a fixation period lasting between 2000 and 12,000 ms. Before beginning the task, participants completed a practice round where they were given step-by-step instructions pertaining to the cue and target conditions as well as 32 practice trials demonstrating the task in the same way as during the actual ANT-R procedure. In total (excluding the practice trials), the ANT-R consist of four blocks, each containing 72 trials (see 47 for further details). The task was performed on a desktop PC, using E-Prime™ software (Psychology Software Tools, Pittsburgh, PA).

### Adult ADHD self-report scale

The ASRS [55] is an 18-item scale where participants are instructed to evaluate the presence and frequency of ADHD-symptoms over the past six months on a Likert scale ranging from never (0) to very often [4]. The scale showed acceptable internal consistency with a Cronbach’s *α* of 0.87. In the present study, this scale was used to describe the symptom severity of the included sample of adults with ADHD at pre-, post- and follow-up sessions.

### Mini international neuropsychiatric interview plus

The M.I.N.I. Plus [54] was administered to assess participants for eligibility for participation as well as to assess the presence of other psychiatric disorders. The interview was administered by an experienced clinical psychologist or by a clinical psychology student with clinical experience—who was supervised by an experienced clinical psychologist.

### Wechsler abbreviated scale of intelligence

Participants completed two subtests from the Wechsler Abbreviated Scale of Intelligence (Matrix reasoning and Vocabulary; [58]) to obtain an estimate of their intelligence quotient.

### Statistical analyses

**Fig. 1:**
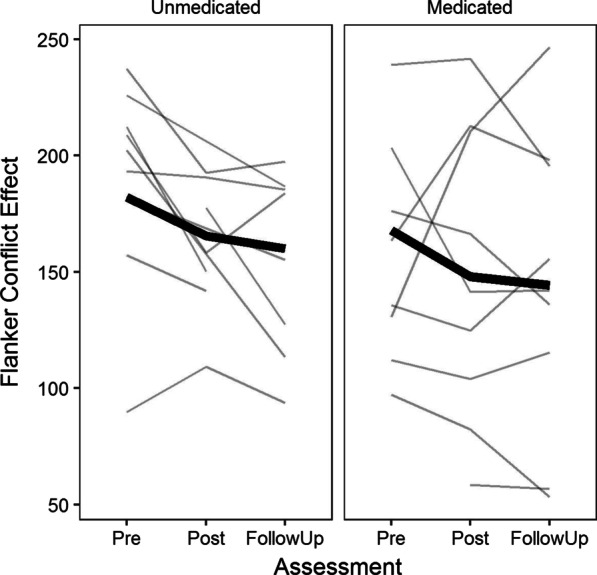
Individual- and group values for the flanker conflict effect (in milliseconds) at each of the three assessments. The bold line depicts changes in group mean across the three assessments, while the regular lines show the development for individual participants

All statistical analyses were conducted using R version 4.0.2 [59] and packages *lme4*, *lmerTest*, *ggplot2, tidyverse* and *SIMR* [60–64]. Linear mixed-effect modeling was used to examine changes in ANT-R scores within individuals over time. Separate models were built for the three dependent variables alerting, orienting and executive control. The baseline models included a random intercept and slope nested within participants. Random intercept and slope for the effects of session were then examined to investigate if this led to improved fit. The covariates of age, sex, IQ and medication use due to ADHD were included as fixed effects and backwards tested. Model fit was assessed by likelihood-ratio tests and covariates were retained if such tests indicated a significant improvement in model fit (See Additional file 1: Table S1). After inclusion of covariates the moderating effects of each predictor on the change in ANT-R measures were examined by adding the interaction between each of these predictors and assessment number to the model. Effect size estimates were calculated using the procedure described by Westfall et al. [65], resulting in estimates approximating Cohen’s *d* [66]. Post-hoc power analyses were conducted on the final models using Monte Carlo simulations as implemented in SIMR. Lastly, results were inspected visually to examine individual patterns of change on the ANT-R score(s) that were shown to be enhanced following GMT. The clinical characteristics of participants showing a negative effect of GMT on ANT-R were further analyzed by inspecting their ASRS scores, medication status, IQ, and comorbid disorders (see Fig. 1).

## Results

### Data preparation

Reaction time data from trials with correct answers were used in the analyses. These were inspected and responses below 100 ms were removed (*N* = 1). Visual inspection indicated that the reaction times (RTs) were not normally distributed. RTs were therefore transformed using an inverse gaussian transformation followed by a multiplication of -1000 as described by Baayen and Milin [67] to approximate a normal distribution. These transformed RTs were then used to calculate measures for the flanker conflict, alerting, and validity effects in line with the descriptions of Fan et al. [47]. After calculation of these measures the data were once again inspected for outliers. For the flanker conflict two extreme trials out of a total of 8167 were removed to ascertain a normal distribution of the data. For the alerting and validity effects extreme values resulted in the removal of eight out of 2771 and one out of 2740 trials, respectively. Furthermore, continuous covariates were centered using grand mean centering. Table 1 shows an overview of the baseline characteristics of the sample as well as information about ANT-R effects and changes in ASRS-scores.

### Linear mixed-effect models

In line with our hypotheses we investigated the changes in the executive control, alerting, and orienting networks. We expected reductions in the flanker conflict and alerting effects across the assessments. Furthermore, we investigated the orienting effect to assess whether participants showed an improvement in the use of spatial cueing.

### Executive control

Following the model fitting approach described under statistical analyses, the final linear-mixed effect model included a random slope and intercept for each individual participant as an effect of assessment-session. Furthermore, the model included fixed effects for session, age and medication status as well as interaction terms for the effects of age by session and medication status by session (AIC 1743.2, see Additional file 1: Table S1 for further information). Monte Carlo simulations based on 1000 repetitions using the final model and an α of 0.05 indicated a power of 78.70% (95% CI: *LL* = 75.03, *UL* = 81.20) for the predictor session.

The results showed a significant fixed effect of assessment-session with reductions in the flanker conflict-effect from baseline to post- and follow up-assessments. The most substantial change occurred between baseline- and post-assessments with a small and non-significant increase from post- to follow up-assessment (*β* = -0.003, *SD* = 0.021, 95% CI = − 0.044 – 0.037, *δt* = − 0.011, *p* = 0.9). The results also showed a significant random effect of assessment-session. Furthermore, results showed negative relationships between the use of medication and the flanker conflict-effect and between age and the flanker conflict effect (See Table 3). The strength of the prediction of the observed values at each assessment-session is visualized in the Additional file 2 : Fig. S1.

**Table 3:** Summary of a linear mixed-effects model of the Flanker Conflict-effect

								Random effects	
Parameter		Fixed effects				By Subject			
	*β*	SE	95% CI	*t*	*p*	δt		*SD*	
			*LL*	*UL*					
Intercept	.458	.030	.399	.518	15.045	< .001***			.122
								Session	
Post-assessment	− .066	.020	− .105	− .026	− 3.286	< .01**	− 0.21		.053
Follow up-assessment	− .069	.027	− .122	− .015	− 2.515	< .05*	− 0.22		.078
Age	− .006	.002	− .011	− .002	− 2.672	< .05*	− 0.02		
Medication status	− .072	.030	− .130	− .014	− 2.423	< .05*	− 0.23		
Post-assessment*Age	.002	.001	− .000	.004	1.700	.11	0.01		
Follow up-assessment*Age	.003	.002	.000	.007	2.040	.06	0.01		
Post-assessment*Medication status	.042	.029	− .014	.099	1.470	.16	0.14		
Follow up-assessment*Medication status	.053	.039	− .023	.129	1.374	.18	0.17		

Age = Age in years centered using grand mean centering, Medication status = Factor describing whether or not participants use ADHD-medication, 95% confidence intervals approximated using the Wald method. *** = *p* < .001, ** = *p* < .01, * = *p* < .05

### Alerting

Results indicated no significant change from baseline to the post- and follow-up assessments (*p* > 0.25), and the model fit was not significantly improved by adding the covariates (all *p-* values > 0.85 compared to the model which only included the fixed effects of session and random effect of participants), as such the original model without covariates was used. For further details see Table 4). Monte Carlo simulations based on 1000 repetitions using the final model and an *α* of 0.05 indicated a power of 17.90% (95% CI: *LL* = 15.57, *UL* = 20.42) for the predictor session.

**Table 4:** Summary of components in a mixed-effects model of the Alerting-effect

								Random effects
*Parameter*		Fixed effects				By Subject		
	β	SE	95% CI	*t*	*p*	δt	*SD*	
			*LL*	*UL*				
Intercept	.091	.013	.066	.117	6.494	< .001***		.033
							Session	
Post-assessment	− .015	.015	− .044	.015	− 0.960	*ns*	− *0.05*	
Follow up-assessment	− .001	.016	− .032	.029	− 0.076	*ns*	− *0.00*	

95% confidence intervals approximated using the Wald method. *** = *p* <.001

### Orienting

There was no significant change from baseline to post-assessment, but a significant effect on the orienting effect from baseline to follow-up, after controlling for the random variance within individual participants (*p* < 0.01. This was also reflected in a significant change between the post and follow up-assessments (*β* = 0.040, *SD* = 0.015, 95% CI = 0.012 – 0.069, *δt* = 0.203, *p* < 0.01). For further details see Table 5. Monte Carlo simulations based on 1000 repetitions using the final model and an *α* of 0.05 indicated a power of 45.00% (95% CI: *LL* = 41.89, *UL* = 48.14) for the predictor session.

**Table 5:** Summary of components in a mixed-effects model of the orienting-effect

								Random effects
*Parameter*		Fixed effects				By Subject		
	β	SE	95% CI	*t*	*p*	δt	SD	
			*LL*	*UL*				
Intercept	.231	.026	.179	.282	8.791	< .001***		.112
							Session	
Post-assessment	.026	.020	− .013	.065	1.293	*ns*	*0.08*	
Follow up-assessment	.063	.028	.007	.119	2.211	< .01***	*0.20*	

95% confidence intervals approximated using the Wald method. *** = *p* <.001, * = *p* <.05

### Visual inspection of individual changes of flanker scores from pre- to follow-up assessments

As can be seen in Fig. 1, the majority of participants showed a decrease in the flanker conflict-effect from baseline to the post-assessment. However, there were relatively large individual differences in change scores (*Range* = [− 98.6 – 79.7 ms]) and a total of four participants showed increases in the flanker conflict-effect from baseline to post-assessment (*Range* = [2.6 – 79.7 ms]). Similarly, five participants showed an increase in the flanker conflict-effect from baseline to follow up-assessment (*Range* = [3.3 – 116.1 ms]). Three of these participants showed an increase from baseline at both timepoints. An inspection showed that all three were men, that two out of the three were older than the average of the sample (51 and 52 years, the third was 33 years), and that the first two had an IQ below the average level of the sample (93 and 103, the third had an IQ of 131). The first two of these participants used medication for ADHD, whereas the last participant did not use such medication. ASRS-scores at baseline were close to the average of the sample (46, 43 and 45, respectively) and increased in parallel with increases in flanker conflict effects for the first two participants at post-assessment (46 to 47 and 43 to 48, respectively) but were greatly reduced for the last participant (45 to 28) despite an increase in the flanker conflict effect. At follow-up all three participants reported changes in ASRS-scores in the same direction as the direction of the flanker conflict-effect relative to post-assessment scores (i.e., the first participant reported an increase [47 to 50], while the last two participants reported reductions [48 to 40, 28 to 26]). The first participant reported ongoing anxiety disorders (panic- and social anxiety disorder) and body dysmorphic disorder, whereas the other two screened positive for antisocial personality disorder.

## Discussion

In the current open trial, we investigated the effects of GMT on executive control and attention allocation as defined by the attention network theory (29) in adults with ADHD. The results supported our expectation that improvements would be found in the executive control network following GMT. However, against our á priori expectation we found no significant changes following GMT on the alerting scores but rather a change in orienting attention scores. The positive change in executive control was detectable immediately after the intervention ended, whereas the change in orienting attention appeared on the follow-up assessment six months later.

The current findings showing improvements in executive control following GMT are in line with previous studies showing that this network is malleable to improvement after non-pharmacological interventions in adults with [42] and without ADHD [45]. The findings are also in accordance with earlier investigations of GMT for other disorders showing a reduction in errors on various neuropsychological measures which include an aspect of response ambiguity [24, 46]. This finding may therefore indicate that changes in goal-management following GMT in adults with ADHD are related to an enhanced ability to handle and detect competing stimuli and thought processes. As such, the current results complement the previous study by Jensen et al. [26] showing specific effects of GMT on executive control in adults with ADHD. Using the ANT-R allowed us to provide support for the notion that this effect on executive control was not driven by improved attention allocation, but a specific effect on conflict detection itself. This demonstrates the advantage of applying experimental paradigms to assess effects of treatment by allowing for investigations of potential mechanisms of change. Another advantage is that the attention network theory is based on a defined neuroscience model of the brain. This may provide indications regarding which brain mechanisms are involved in for instance improvement of executive control following GMT [23, 25, 26]. The executive control network has been shown to rely on frontal brain regions in several fMRI studies [68–70]. Among the regions involved in the executive control network are the anterior cingulate cortex (ACC) and insula, regions that are also part of the salience network and involved in conflict monitoring and upregulation of cognitive control in response to uncertainty or error [71, 72]. In accordance with the assumptions underlying GMT, a possible explanation would therefore be that GMT acts through changes in brain networks involved in executive control and orienting/alertness [20]. Importantly, activation in the ACC and insula has been shown to differentiate between individuals with ADHD and non-ADHD controls during performance of cognitive inhibition tasks [73]. Improvements in the connectivity of the ACC has also been shown to be associated with symptom remittance among adolescents with ADHD [74]. As findings from studies of various populations, including ADHD, indicate that both short-term mindfulness interventions [75–78] and cognitive rehabilitation interventions [79, 80] may improve the functional connectivity of the ACC and/or insula, this may also be a possible mechanism involved in our findings. Future studies may delineate the importance of these components for functional changes following GMT.

The adults with ADHD did not show the expected improvement in alertness after GMT. Rather, and in contrast to our expectations, they showed significant changes in results on the measure of the orienting network at follow up. Interestingly, these findings are in accordance with previous studies of mindfulness training showing effects on executive control and orienting after shorter periods of training [44, 45]. It is important to note that the attention networks are believed both to interact and to operate independently from each other. This is in line with the view emphasizing the importance of also studying supportive processes such as attention allocation in order to improve the understanding of conflict detection [81]. For instance, there may be an overlap of functioning between the orienting attention of ANT-R when defined by validity of cues and executive control since they rely on some of the same brain regions (i.e., ACC and insula; 47). This was supported in a study by Trautwein et al. [70] in that invalidly cued targets induced activation in the ACC and insula. We have also found in a previous study that the interaction between early updating of stimuli (e.g., orienting attention) and conflict detection (e.g., executive control) associated with higher flexibility of the autonomous nervous system (i.e., heart rate variability; 82). Similarly, being alert to *when* stimuli appear on the screen may also affect an alertness to *where* the same stimuli appear (see 28). An improvement in the orienting attention and/or the executive control network may therefore reflect higher alertness following GMT despite this not being overtly observed through higher alerting scores following GMT.

In addition to the traditional focus on treatment effects on a group level, it is of clinical relevance to look at individual change scores to investigate if some participants may have a negative effect of GMT. Our sample was too small to statistically test differences between the three men who, on visual inspection of the flanker conflict score (i.e., executive control network score), appeared to have a negative effect by being less efficient on both assessments after GMT compared to baseline. However, by looking at other variables/scores of clinical interest from the assessments of these three male participants, we observed that with the exception of sex and that two out of three had a comorbid antisocial personality disorder (according to the M.I.N.I. Plus), there did not appear to be any specific variable included in our study in which these participants systematically differed from the rest of the sample and that could explain the differential effect of GMT on ANT-R. However, of interest, their negative change on the flanker conflict score for the most part mirrored changes in ADHD symptom reports on ASRS after GMT. It is challenging to delineate if this negative development was due to lack of effect of GMT or if something else occurred in these participants’ lives that prevented them from engaging in the remediation practices of GMT. Few studies show such individual effect scores of GMT or of other types of treatment. This can be an important way to improve the understanding of which participants experience both positive and negative effects, and thereby improve individually targeted treatment in patient groups.

## Strengths, limitations, and future directions

We believe that the current results support the use of attention network theory and the ANT-R in future studies of interventions for ADHD and in investigations of GMT, and that such studies may provide important insights into potential mechanisms involved in reducing symptoms and impairments associated with the disorder being studied. Furthermore, the recruitment of a clinically-based sample of adults with ADHD probably increased the transferability of the results to patients with ADHD seeking treatment in the health care system. Also, the description of individual trajectories and the attempts to characterize individual differences in effects of the intervention can be an inspiration for adapting the same approach in future treatment studies.

There are several limitations to the current study. First of all, due to the lack of a control group, practice effects cannot be ruled out. We do, however, believe that the differential effects on the separate networks and the differing timing of effects speak against such an interpretation. Investigations of practice effects in Flanker-tasks also seem to indicate that these are relatively small and quite equal between congruent and incongruent trials [83, 84]. Secondly, the sample size was small and power analyses only indicate that the model investigating executive control achieved statistical power close to what is satisfactory. The sample size also did not allow for statistical power to perform between-group analyses to explore the influence of factors such as medication status and ADHD-subtype. Earlier findings indicate that these factors may influence RTs on the ANT-R (e.g., [42]) and we found an indication in the current study of a negative relationship between medication use and the flanker effect. Related to this, the fact that analyses were limited to participants who completed two or more assessments may have influenced the finding. As the current sample consisted of adults with ADHD that were mainly recruited from local outpatient clinics, many of the participants also had comorbid disorders, mainly anxiety disorders. It is therefore possible that the current results may also reflect effects not directly related to ADHD but to concurrent anxiety disorders or other comorbid conditions. Finally, the current sample attained above average estimates of full scale-IQ. As such, it is possible that the reported results and/or the attained effect of the intervention may not be generalizable to samples with lower intelligence.

We argue that the current results indicate that there is reason to believe that GMT may be an advantageous intervention for adults with ADHD and that this should be evaluated in a larger randomized trial. Furthermore, we believe that inclusion of brain imaging techniques in such a trial could clarify the current results and inform the validity of the possible explanations discussed above.

## Conclusion

The current open trial found an improvement in one aspect of attention, namely executive control, following GMT in a sample of adults with ADHD. Our finding thus suggests that GMT may be an efficient intervention for ADHD and further, that improvements in executive control may be a potential mechanism of change for adults with ADHD using this training procedure. A possible brain-correlated mechanism explaining this improvement may be enhanced functioning or connectivity in the ACC and/or insula following the intervention. Future randomized controlled studies of GMT are encouraged to include the ANT-R in combination with brain imaging techniques such as fMRI to increase the neurobiological understanding of effects of GMT in ADHD and other patient groups.

## Supplementary Information

**Additional file 1: Table S1:** Summary of linear mixed-effects model comparisons for the Flanker Conflict-Effect.

**Additional file 2: Fig. S1: **A graphical illustration of observed vs predicted flanker conflict effects for each individual participant

## Data Availability

The datasets generated and analyzed during the current study are not publicly available due to limitations in the ethical approval for the study but are available from the corresponding author on reasonable request.

## Abbreviations

ACC: Anterior cingulate cortex
ADHD: Attention-deficit/hyperactivity disorder
ANT: Attention network test
ANT-R: Attention network test-revised
ASRS: Adult ADHD self-report scale
DSM-IV: Diagnostic and statistical manual of mental disorders, 4th edition
DSM-5: Diagnostic and statistical manual of mental disorders, 5th edition
ECG: Electrocardiogram
GMT: Goal management training
ICD-10: International classification of diseases
M.I.N.I. Plus: Mini international neuropsychiatric interview plus
RT: Reaction time
