# Supplementary material for: Goal management training improves executive control in adults with ADHD: an open trial employing attention network theory to examine effects on attention
Source: BMC Psychol. 2022 Aug 26;10:207. doi: 10.1186/s40359-022-00902-9 (PMC9414421; doi:10.1186/s40359-022-00902-9)
Supplement: Supplementary file 1 — Additional file 1: Table S1: Summary of linear mixed-effects model comparisons for the Flanker Conflict-Effect. [file 40359_2022_902_MOESM1_ESM.docx]

| Supplementary table 1: Summary of linear mixed-effects model comparisons for the Flanker Conflict-Effect | | | | | | | | | |
| --- | --- | --- | --- | --- | --- | --- | --- | --- | --- |
|  |  | Model description | |  | Test against previous model | | | | |
| Model |  | Fixed-effects | Random-effects |  | AIC | ΔAIC |  | Statistic | *p* |
| Model 0 |  | None | By participant random intercepts |  | 1888.5 |  |  |  |  |
| Model 1 |  | Assessment | Model 0 + by-assessment random contrast and intercepts. |  | 1744.8 | -143.7 |  | χ^2^(7) = 157.7 | <.001 |
| Model 2 |  | Assessment + age, sex, IQ, & medication status | Model 1 |  | 1746.3 | +1.5 |  | χ^2^(4) = 6.54 | .162 |
| Model 3 |  | Assessment + age, IQ, & medication status | Model 1 |  | 1744.4 | -1.9 |  | χ^2^(1) = 0.09 | .765 |
| Model 4 |  | Assessment + age, & medication status | Model 1 |  | 1743.2 | -1.2 |  | χ^2^(1) = 0.78 | .378 |
| Model 5 |  | Assessment + age | Model 1 |  | Model failed to converge | | | | |
| Model 6 (final model) |  | Assessment + age, medication status, & interaction terms for assessment by age and assessment by medication status | Model 1 |  | 1743.2 | 0.0 |  | χ^2^(1) = 2.10 | .147 |
