# Supplementary material for: Goal management training improves executive control in adults with ADHD: an open trial employing attention network theory to examine effects on attention
Source: BMC Psychol. 2022 Aug 26;10:207. doi: 10.1186/s40359-022-00902-9 (PMC9414421; doi:10.1186/s40359-022-00902-9)
Supplement: Supplementary file 2 — Additional file 2: Fig. S1: A graphical illustration of observed vs predicted flanker conflict effects for each individual participant [file 40359_2022_902_MOESM2_ESM.docx]

Supplementary Figure 1: A graphical illustration of observed vs predicted flanker conflict effects for each individual participant


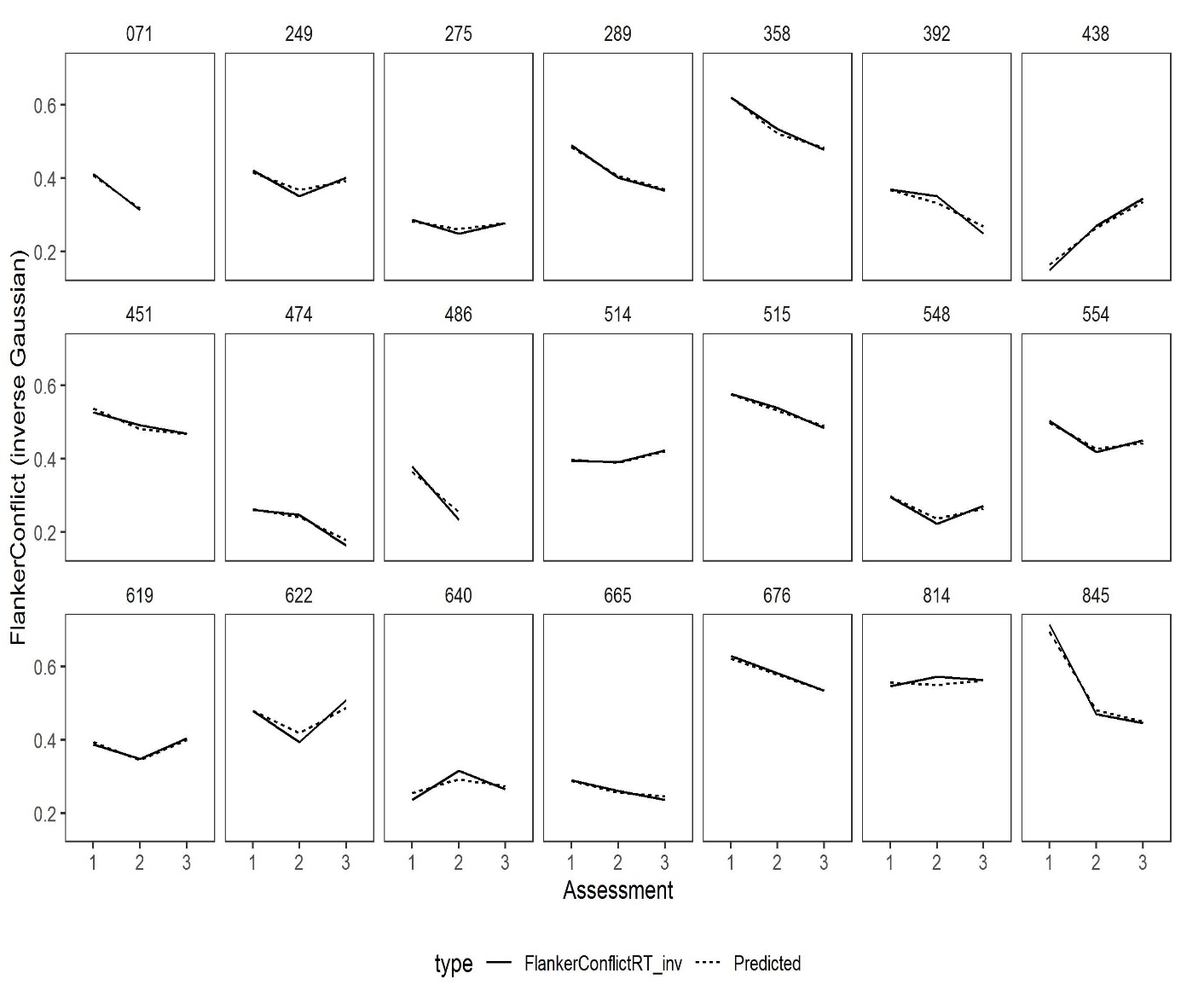


^Note: The figure depicts comparisons between observed values (complete lines) and predicted values based on the final model (dashed lines).^
